# Age Differences in the Neuroelectric Adaptation to Meaningful Sounds

**DOI:** 10.1371/journal.pone.0068892

**Published:** 2013-07-25

**Authors:** Ada W. S. Leung, Yu He, Cheryl L. Grady, Claude Alain

**Affiliations:** 1 Department of Occupational Therapy and Centre for Neuroscience, University of Alberta, Edmonton, Canada; 2 Rotman Research Institute, Baycrest Centre for Geriatric Care, Toronto, Ontario, Canada; 3 Department of Psychology, University of Toronto, Ontario, Canada; 4 Department of Psychiatry, University of Toronto, Ontario, Canada; 5 Institute of Medical Sciences, University of Toronto, Ontario, Canada; National University of Singapore, Singapore

## Abstract

Much of what we know regarding the effect of stimulus repetition on neuroelectric adaptation comes from studies using artificially produced pure tones or harmonic complex sounds. Little is known about the neural processes associated with the representation of everyday sounds and how these may be affected by aging. In this study, we used real life, meaningful sounds presented at various azimuth positions and found that auditory evoked responses peaking at about 100 and 180 ms after sound onset decreased in amplitude with stimulus repetition. This neural adaptation was greater in young than in older adults and was more pronounced when the same sound was repeated at the same location. Moreover, the P2 waves showed differential patterns of domain-specific adaptation when location and identity was repeated among young adults. Background noise decreased ERP amplitudes and modulated the magnitude of repetition effects on both the N1 and P2 amplitude, and the effects were comparable in young and older adults. These findings reveal an age-related difference in the neural processes associated with adaptation to meaningful sounds, which may relate to older adults’ difficulty in ignoring task-irrelevant stimuli.

## Introduction

Auditory adaptation refers to the reduction of neural responses when a sound is heard repetitively within a period of several seconds [1,2]. It is thought to reflect a reduction of sensitivity in the auditory system to repeated stimuli and enhance sensitivity to novel stimuli [3]. From a physiological perspective, neural adaptation reflects a process by which the brain encodes stimulus invariance and likely occurs because of neural refractoriness in which a neuron can only respond to a stimulus after a sufficient period of recovery following a response to a preceding stimulus [4,5]. Studies using event-related potentials (ERPs) have consistently reported a reduction in the amplitude of a negative wave peaking at about 100 ms after sound onset (N1) with stimulus repetition [6,7,8]. Other studies have shown reduced P2 amplitudes (positive wave peaking at about 180 ms post-stimulus) for sounds coming from identical spatial locations [9]. These patterns of neural adaptation have been related to increased cognitive efficiency, such as improved stimulus identification [10], auditory memory [11], and rapid learning [7].

Evidence from ERP studies suggest that aging may be associated with deficits in inhibitory control (e.g., [12,13,14]) and/or impaired sensory memory [15,16,17,18]. Studies have also found that older adults, relative to young adults, show smaller repetition effects in the electrophysiological responses to repeated tones within trials [19,20]. Recently, Grady et al. [21] tested neural adaptation to complex meaningful sounds (e.g., dog barking, phone ringing etc) using functional magnetic resonance imaging (fMRI), and found that aging was associated with reduced adaptation to repetitions of sound location in parietal cortex and reduced adaptation to repetitions of sound identity in anterior temporal cortex.

To date, studies that focused on ERP adaptation have generally used pure tones (e.g., [22,23,24]). While these studies provide insight into the representation of acoustic features (e.g., pitch), they tell us little about the effect of age on representations of more complex sound objects. In the current study, we measured ERP while young and older adults listened to a series of four complex, everyday sounds that were either identical or differed in their spectro-temporal characteristic and/or spatial location. This design allowed us to determine both domain-specific and domain non-specific aspects of neuroelectric adaptation. The domain-specific adaptation refers to the adaptation resulting from repetition of either the sound content, i.e., spectro-temporal characteristic of sounds (identity domain), or sound location, i.e., spatial characteristic of sounds (spatial domain). The domain non-specific adaptation refers to the adaptation resulting from repetition of both the sound content and sound location in a stimulus train, i.e., the conjunction of identity and spatial domains. In addition, we included an experimental manipulation that examined the influence of simulated MRI noise to determine whether the effect of this noise on neural adaptation might have influenced the results from Grady et al. [21]. We hypothesized that age would interact with the domain-specific aspects of neural adaptation, and that noise would reduce, but not eliminate, neural adaptation in young and older adults.

## Methods

### Ethics Statement

The present study was approved by the Research Ethics Board of the Toronto Academic Health Sciences Network and the University of Toronto and Baycrest Centre Human Subject Review Committees. Participants gave written informed consent before participating in the study and were paid for their participation in the experiment.

### Participants

Twelve young adults (mean age ± standard deviation (SD), = 25.2 ± 3.9; 5 men) and twelve older adults (69.7 ± 7.2 yrs; 5 men) volunteered to participate in this study. Participants were recruited from the local community and laboratory personnel at the Rotman Research Institute, Baycrest Centre. They were screened to exclude health problems and/or medications that might affect cognitive function and brain activity. All participants had pure-tone thresholds less than or equal to 20 dB hearing level (HL) in the range of 250-2000 Hz in both ears. Older adults scored in the normal range, between 27 and 30 (mean 29.08, standard error (SE) = 0.79), on the Mini-Mental State Examination which is a brief 30-point questionnaire test commonly used to screen for cognitive impairment [25].

### Stimuli

Stimuli consisted of everyday sounds from four categories: human non-speech sounds (e.g., a man laughing, a baby crying), animal sounds (e.g., a rooster crowing, a dog barking), musical sounds (e.g., a cello being played, a piano being played), and manmade objects (e.g., a door bell ringing, a car engine running). The sounds were chosen from a large databank (http://www.findsounds.com/types.html) and only those that could be unambiguously assigned (by a group of individuals not included in the current report) to the appropriate category were used in the study. Altogether, one hundred and ten sounds were selected for use in this study. Among them, there were twenty-eight human non-speech sounds, twenty-seven animal sounds, twenty-seven musical sounds and twenty-eight manmade object sounds. The auditory stimuli were edited to have a duration of 1005 ms that included an onset and offset ramp shaped by two halves of an 8-ms Kaiser window. All of the stimuli had the same averaged root mean square (RMS) power across the entire duration of the sound (i.e., 1005 ms). Additionally, total RMS power within 40 ms of the sound onset, which is within the temporal integration of the N1 wave [26], was analyzed for each sound. The means of the total RMS power for the human non-speech, animal, musical and manmade objects sounds were 20.52 dB (SD = 5.61), 19.49 dB (SD = 6.26), 17.80 dB (SD = 7.43) and 17.00 dB (SD = 6.26) respectively and were not statistically different, *F*(3,109) = 1.718, *p* = 0.28. This indicates that the acoustic energy for the first 40-ms was comparable between the four sound categories.

Stimuli were digitally generated at a sampling rate of 24,414 Hz using a System 3 Real-Time Processor from Tucker Davis Technologies (TDT) (Alachua, FL). The stimuli were fed into a headphone driver (TDT HB-7) and then presented to participants via a pair of headphones (Sennheiser HD 256) at 68 dB sound pressure level (SPL). The intensity of the stimuli was measured using a Larson-Davis SPL meter (Model 824, Provo, Utah). The sounds were calibrated using a 2 cylindrical cavity (cc) coupler on an artificial ear (Model AEC 100). During the calibration process, the Sennheiser HD 265 headphones were placed over the 2 cc coupler, which were connected to the SPL meter, and separate measurements were taken for left and right ear channels. A head-related transfer function (HRTF) was used to present the stimuli at four virtual locations along the azimuth: +30°, -30°, +95°, -95°. A variety of HRTF coefficients were tested and those that resulted in the most accurate responses during an initial discrimination test of four different locations were used for each participant.

**Figure 1:**
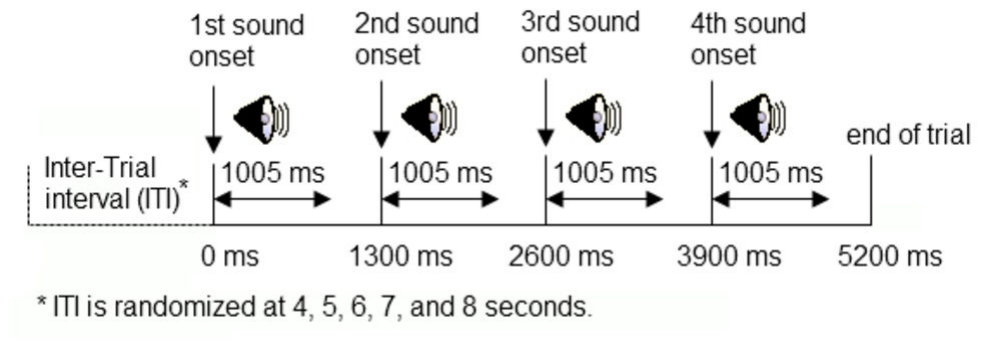
A schematic diagram showing four consecutive sound stimuli in a trial.

Stimuli were presented using a modified version of a procedure reported by Goh et al. [27], in which each trial consisted of four sounds, presented at a constant stimulus onset asynchrony of 1300 ms (Figure 1). This design takes advantage of the fact that immediate repetitions produce larger adaptation than delayed repetitions [2], so it should be maximally sensitive to age differences, but at the same time allows intermix of trial types in an ERP paradigm. The trials were presented with inter-trial intervals (ITI) that varied randomly between 4 and 8 seconds (500 ms step, rectangular distribution). There were four trial types: same sound, same location (S_S_S_L_); same sound, different location (S_S_D_L_); different sound, same location (D_S_S_L_); and different sound, different location (D_S_D_L_). For the trial types D_S_S_L_ and D_S_D_L_, the four stimuli within the sequence were from different sound categories.

To study the impact of noise on adaptation, participants were presented with two different listening conditions. In the no noise condition, participants were presented with the four different trial types in random order without background noise. In the noise condition, the same stimuli were presented against simulated noise from a 3T MR scanner that was presented at 90 dB SPL through a GSI audiometer and delivered via two speakers located at about 1 meter from the left and right (at a 45^o^ angle) of the participant. A total of 240 trials were presented to participants (30 trials per trial type per noise condition). The order of conditions was counterbalanced between participants. The intensity of the noise stimuli was measured using a Larson-Davis SPL meter with a free field microphone (Larson-Davis Model 2559) placed at the same position where the participants would be sitting. During the experiment, participants sat comfortably in a chair in a sound-attenuated chamber and were asked to listen passively to the sounds (no response required). They were told to keep their eyes open and to look straight ahead. Participants’ behavior was monitored using a closed-circuit video camera during EEG recording, and were given verbal warning and a chance to stretch if they closed their eyes for more than a few seconds or appeared drowsy.

### Data Acquisition and analysis

The electroencephalogram was digitized continuously (sampling rate 500 Hz per channel) from an array of 64 electrodes with a bandpass of 0.05–100 Hz using NeuroScan Synamps2 (Compumedics, El Paso, TX, USA). Eye movements were monitored with electrodes placed at the outer canthi and at the inferior orbits. During recording, all electrodes were referenced to the midline central electrode (Cz); for off-line data analysis, they were re-referenced to an average reference. For each participant, a set of ocular movements was obtained prior to and after the experiment [28]. From this set, averages were calculated for eye-blinks and both lateral and vertical eye movements. A principal component analysis of these averaged recordings provided a set of components that best explained the eye movements. The scalp projections of these components were then removed from the experimental ERPs in order to minimize ocular contamination, using Brain Electrical Source Analysis (BESA 5.2.0). Epochs contaminated by excessive peak-to-peak deflection (±100 µV) after correcting for ocular contaminations were excluded from the averages. For each participant, the remaining epochs were averaged according to electrode site, noise condition, and trial type using BESA. The ERPs were digitally filtered to attenuate frequencies above 30 Hz (12 dB/Oct; zero phase).

The effect of age on the ERP adaptation was investigated by analyzing peak amplitudes. The analysis epoch consisted of 200 ms of pre-stimulus activity and 5000 ms of post-stimulus activity. This epoch duration was chosen to encompass the four sound objects within each trial thereby allowing us to examine transient onset responses elicited by each sound within the trial. Peak amplitudes were determined as the largest positivity or negativity during a specific interval. The measurement intervals were 80-180 ms (N1), and 180-300 ms (P2) post-stimulus onset for each stimulus in the trial.

We analyzed the patterns of neural adaptation across the four sound stimuli using a mixed model repeated measures ANOVA with age as the between-groups factor, and noise condition (i.e., noise, and no noise), trial type (i.e., D_S_D_L_, D_S_S_L_, S_S_D_L_, and S_S_S_L_), position in the sequence (i.e., 1^st^, 2^nd^, 3^rd^ or 4^th^) and electrode (i.e., FCz, FC1, FC2, Cz, C1, and C2) as the within-group factors for each of the N1 and P2 waves. We used a cluster of electrodes at fronto-central sites where the N1 and P2 wave amplitudes are typically the largest and because it provides a good estimate of ERP amplitude while taking into account individual differences in amplitude distribution. When appropriate, the degrees of freedom were adjusted with the Greenhouse-Geisser epsilon (ε) and all reported probability estimates were based on the reduced degrees of freedom, although the original degrees of freedom were reported. This analysis provided information about the difference in the patterns of neural adaptation between young and older adults, taking into account each individual sound in the stimulus train. We also examined domain-specific and domain non-specific adaptation by analyzing the interaction effects between trial type and position.

To further determine domain specificity of neural adaptation, we compared the magnitude of N1 and P2 amplitude changes across the four stimulus types in young and older adults. We subtracted the N1 and P2 peak amplitude of the fourth sound from the first sound within the train in each of the four trial types, i.e., D_S_D_L_, D_S_S_L_, S_S_D_L_, and S_S_S_L_. For N1 amplitudes, the more negative the value the greater the magnitude of amplitude decline; for P2 amplitude, the more positive the value the greater the magnitude of amplitude decline. In this part of the analysis, repeated measures ANOVAs with age as the between-groups factor, and noise condition (i.e., noise, and no noise), trial type (i.e., D_S_D_L_, D_S_S_L_, S_S_D_L_, and S_S_S_L_) and electrode (i.e., FCz, FC1, FC2, Cz, C1, and C2) as the within-group factors were computed for each of the N1 and P2 waves. This analysis allowed a more precise comparison on the change of amplitudes from the beginning to the end of each trial, without being statistically weakened by the number of positions and the variation of peak amplitudes for stimuli in the intermediate positions. We expect to find domain-specific adaptation with significant differences between D_S_D_L_ and D_S_S_L_ and/or S_S_D_L_ trials, and domain non-specific adaptation with a significant difference between D_S_D_L_ and S_S_S_L_ trials.

## Results

**Figure 2:**
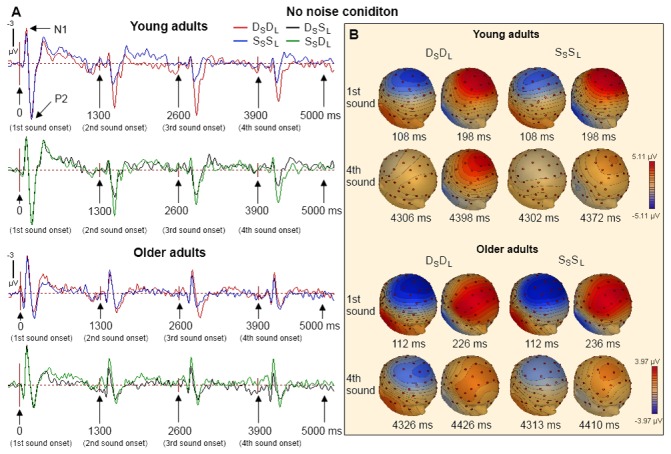
ERP waves elicited by four consecutive sound stimuli in the D_S_D_L_, D_S_S_L_, S_S_D_L_ and S_S_S_L_ trials in the no noise condition. (A) Evoked responses averaged for young and older adults at Cz in the D_S_D_L_, D_S_S_L_, S_S_D_L_ and S_S_S_L_ trials. (B) The topographic maps of the N1 and P2 waves at latencies showing peak ERP amplitudes at Cz for the 1^st^ and 4^th^ sound in the D_S_D_L_, and S_S_S_L_ trials among young and older adults.

**Figure 3:**
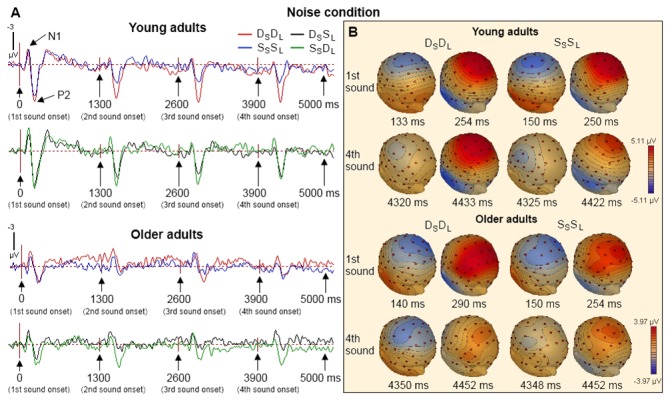
**ERP waves elicited by four consecutive sound stimuli in the D_S_D_L,_ D_S_S_L_, S_S_D_L_ and S_S_S_L_ trials in the noise condition**. (A) Evoked responses averaged for young and older adults at Cz in the D_S_D_L,_ D_S_S_L_, S_S_D_L_ and S_S_S_L_ trials. (B) The topographic maps of the N1 and P2 waves at latencies showing peak amplitudes at Cz for the 1^st^ and 4^th^ sound in the D_S_D_L,_ and S_S_S_L_ trials among young and older adults.

Figures 2 and 3 show the group average ERPs from the young and older adults on each of the four trial types in the condition without and with noise, respectively. Each sound within the sequence generated large N1 and P2 waves that peaked at about 120 and 190 ms after sound onset, respectively. The effects of age, noise condition, trial type, and sound position on the N1 and P2 wave amplitudes were examined separately. There were no clear N1 subcomponents such as the N1a and N1c and consequently no further analysis was done on the N1 subcomponents.

### Effect of age on the adaptation of N1 amplitude

**Figure 4:**
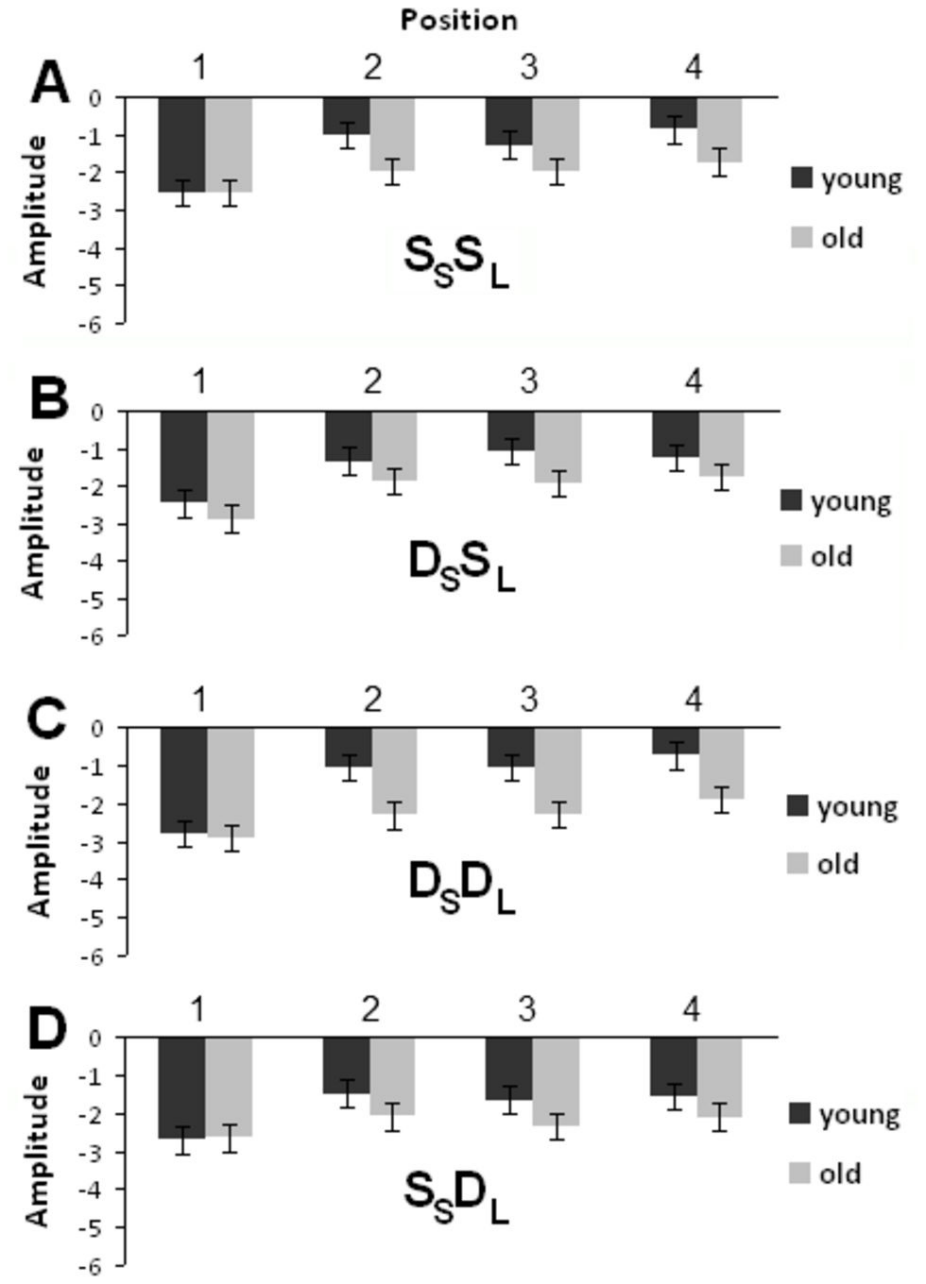
Plot of N1 peak amplitudes across the four positions regardless of noise. (A) S_S_S_L_ trials. (B) D_S_S_L_ trials. (C) D_S_D_L_ trials. (D) S_S_D_L_ trials. Error bars indicate standard error of the mean.

Repeated measures ANOVA revealed main effects of age, *F*(1,22) = 4.363; *p* = 0.049, noise condition, *F*(1,22) = 17.415; *p* < 0.001, and position, *F*(3,66) = 14.912; *p* < 0.001, and a significant interaction effect between noise condition and position, *F*(3,66) = 16.409; *p* < 0.001. This interaction was caused by markedly reduced N1 amplitudes from the first to the second sound of the sequence when the stimuli were not embedded in noise (Figures 2 and 3). The main effect of age on N1 amplitude could be partly related to age differences in audiometric thresholds. To assess this possibility, the effects of age on N1 were re-analyzed using the mean audiometric thresholds for octave frequencies between 250 and 2000 Hz from both ears as a covariate in the repeated measures ANOVA. This analysis also yielded a main effect of age, *F*(1,21) = 7.823; *p* = 0.011. Other main and interaction effects obtained with the covariate were also comparable with that obtained without the covariate. These included significant main effects of noise condition, *F*(1,21) = 3.819; *p* = 0.064, and position, *F*(3,63) = 8.954; *p* < 0.001, and a significant interaction effect between noise condition and position, *F*(3,63) = 3.892; *p* = 0.013. These results indicated that the main effect of age on N1 may not be accounted for by age differences in audiometric thresholds alone. Trend analysis revealed significant linear and quadratic trends for sound position, *F*(1,22) = 18.407 and 10.437; *p* = 0.001 and 0.004, respectively (Figures 2, 3, and 4).

### Effect of age on the adaptation of P2 amplitude

**Figure 5:**
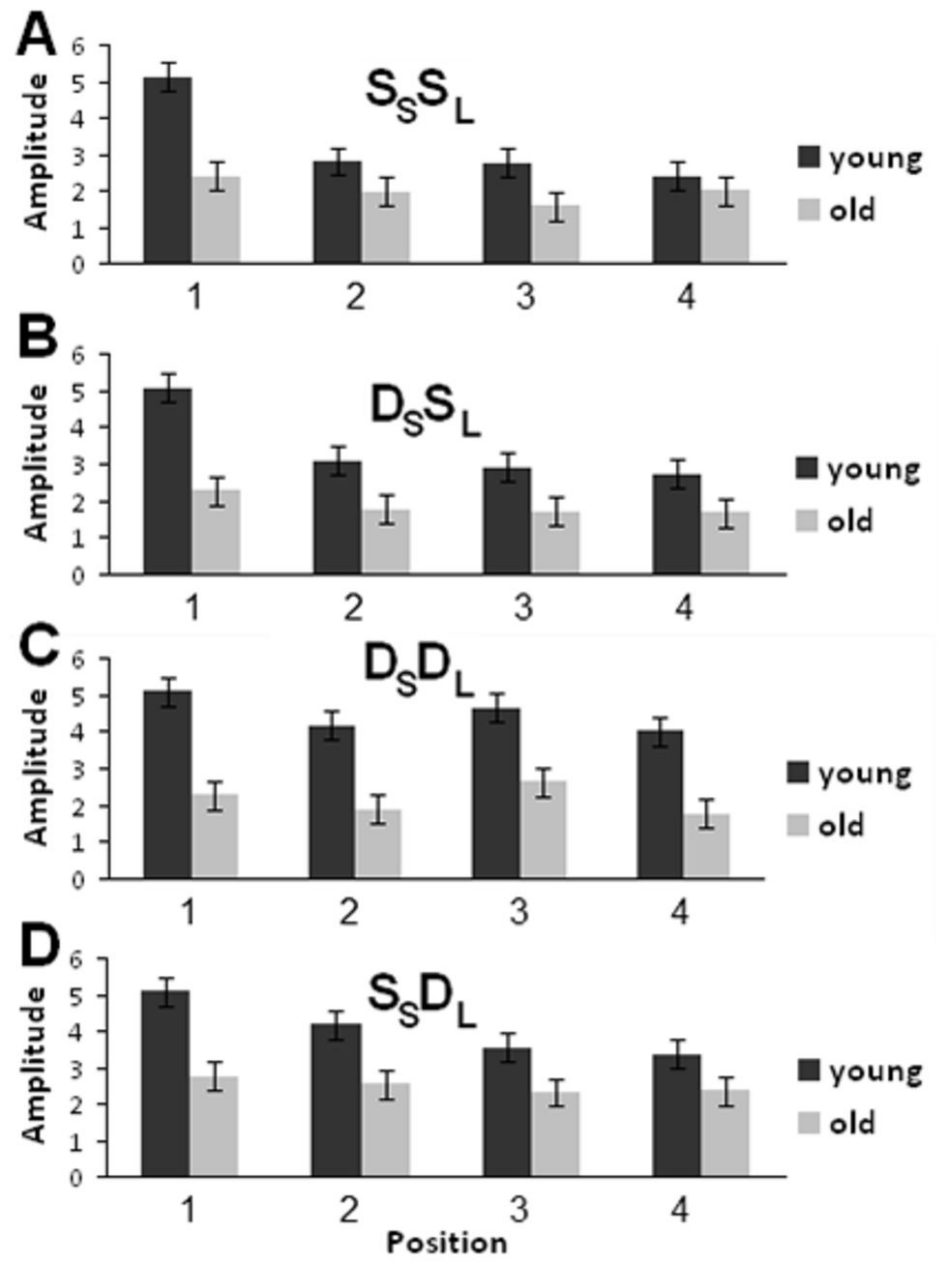
Plot of P2 peak amplitudes across the four positions regardless of noise. (A) S _S_S_L_ trials. (B) D_S_S_L_ trials. (C) D_S_D_L_ trials. (D) S_S_D_L_ trials. Error bars indicate standard error of the mean.

Consistent with prior research [29,30], we found an age-related decrease in P2 amplitude, *F*(1,22) = 8.047; *p* = 0.01. The main effect of age remained even after controlling for age differences in hearing sensitivity using the mean audiometric thresholds as a covariate in an analysis of covariance, *F*(1,21) = 4.357; *p* = 0.049. Repeated measures ANOVA also revealed main effects of trial type, *F*(3,66) = 12.953; *p* < 0.001, and sound position, *F*(3,66) = 14.584; *p* < 0.001. Pairwise comparison showed that the P2 amplitudes of the D_S_D_L_ and S_S_D_L_ trials were greater than that of the S_S_S_L_ and D_S_S_L_ trials, *ps* < 0.05, and the P2 amplitudes for the first sound of the sequence was significantly greater than that of all other sound stimuli in the train (*ps* < 0.05) (Figures 2, 3, and 5). The effect of noise was not significant nor was the interaction between noise and age, *Fs* < 1.6. However, there were significant interaction effects between trial type and position, *F*(9,198) = 5.356; *p* < 0.001, and between age, trial type, and position, *F*(9,198) = 1.924; *p* = 0.05. Trend analysis also revealed a significant interaction between age, trial type, and position on the quadratic trend, *F*(1,22) = 5.122; *p* = 0.034. To better understand these interaction effects, we performed separate ANOVAs in young and older adults.

In young adults, repeated measures ANOVA (trial type × position) revealed significant main effects of trial type, *F*(3,33) = 10.500; *p* < 0.001, and position, *F*(1,33) = 17.980; *p* < 0.001. There was a significant interaction effect between trial type and position, *F*(9,99) = 3.638; *p* = 0.001. For both the S_S_S_L_ and D_S_S_L_, the main effects of position were significant, *F*(3,33) = 21.638 and 8.905, respectively; *p* < 0.001 in both cases. The sound at the 1^st^ position generated larger P2 amplitudes than the subsequent stimuli, *ps* < 0.01. The linear and quadratic trends were also significant, *Fs*(1,11) > 6.558; *ps* < 0.026, in both cases. The P2 amplitudes decreased from the first to the second position and showed little change in amplitude in the subsequent positions (Figures 2, 3, and 5A,B). For the S_S_D_L_ trials, the main effect of position was significant, *F*(3,33) = 9.370; *p* < 0.001, and the sound at the 1^st^ position generated larger P2 than the 2^nd^, 3^rd^ and 4^th^ positions, *ps* < 0.05 (Figures 2, 3, and 5D). In contrast to the S_S_S_L_ and D_S_S_L_ trials, the S_S_D_L_ trials showed only a linear trend, *F*(1,11) = 24.309; *p* < 0.001, indicating a gradual decrease of P2 amplitudes from the first to the forth position (Figure 5D). For the D_S_D_L_ trials, the main effect of position was significant, *F*(3,33) = 3.025; *p* = 0.047, and the P2 amplitude was greater at the 1^st^ than the 4^th^ position (Figures 2, 3 and 5C). The linear trend approached significance, *F*(1,11) = 3.961; *p* = 0.072) (Figure 5C). In older adults, the main effect of position was marginally significant, *F*(3,33) = 2.901, *p* = 0.05, and the P2 amplitude was greater at the 3^rd^ than the 4^th^ position (*p* = 0.049). The main effect of noise was significant, *F*(1,11) = 7.396; *p* = 0.02, and the P2 amplitude was larger in the no noise than in the noise condition. The interaction between noise, trial type, and position was not significant, *F* < 1.5. In order to ensure that this reduced P2 adaptation was not simply due to small P2 amplitude in older adults, a separate ANOVA was performed using only the fronto-central electrodes (i.e., FCz, FC1 and FC2) where the P2 wave was largest in older adults. The analysis revealed main effects of position, *F*(3,33) = 5.027; *p* = 0.006, and noise, *F*(1,11) = 6.990; *p* = 0.023. The interaction between noise, trial type, and position was not significant, *F* < 1. These results showed that the P2 at frontal sites was more sensitive to change in older adults, which suggested that the reduced P2 adaptation in older adults was not simply the consequence of reduced P2 amplitude or the floor or ceiling effects.

### Magnitude of neural adaptation in young and older adults

**Figure 6:**
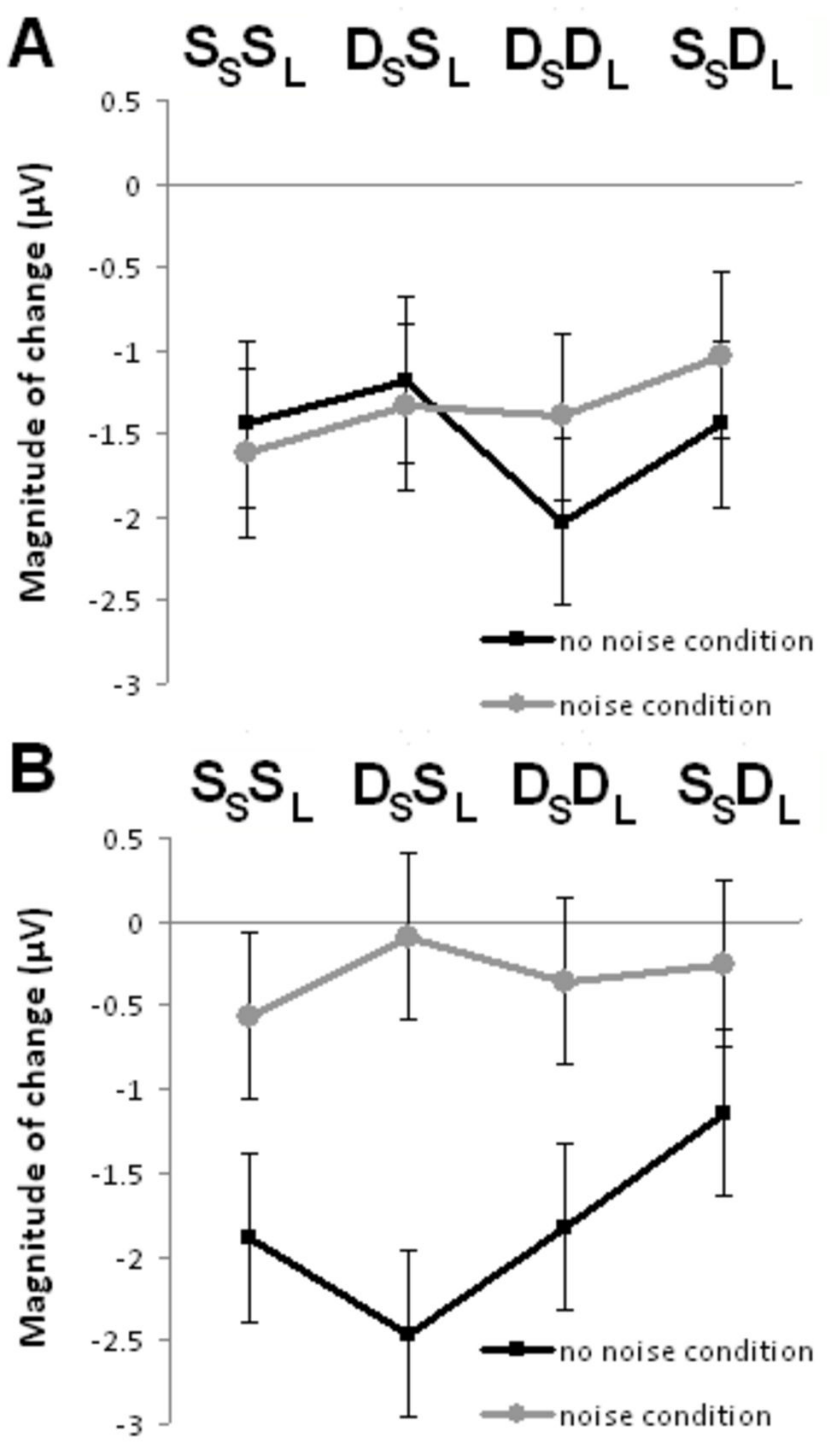
Plot of the magnitude of N1 amplitude changes from the 1^st^ to 4^th^ position across the four trial types in the absence and presence of noise. (A) Young adults. (B) Older adults. Error bars indicate standard error of the mean.

For the N1 wave, repeated measures ANOVA (age × noise condition × trial type) revealed a significant main effect of noise condition, *F*(1,22) = 6.632; *p* = 0.017, and an interaction effect between age and noise condition, *F*(3,66) = 3.002; *p* = 0.037). Older adults showed smaller neural adaptation in the noise compared to no noise condition, *F*(1,11) = 31.400; *p* < 0.001 (Figure 6B). No significant findings were obtained for young adults, *F*s < 1.4 (Figure 6A).

**Figure 7:**
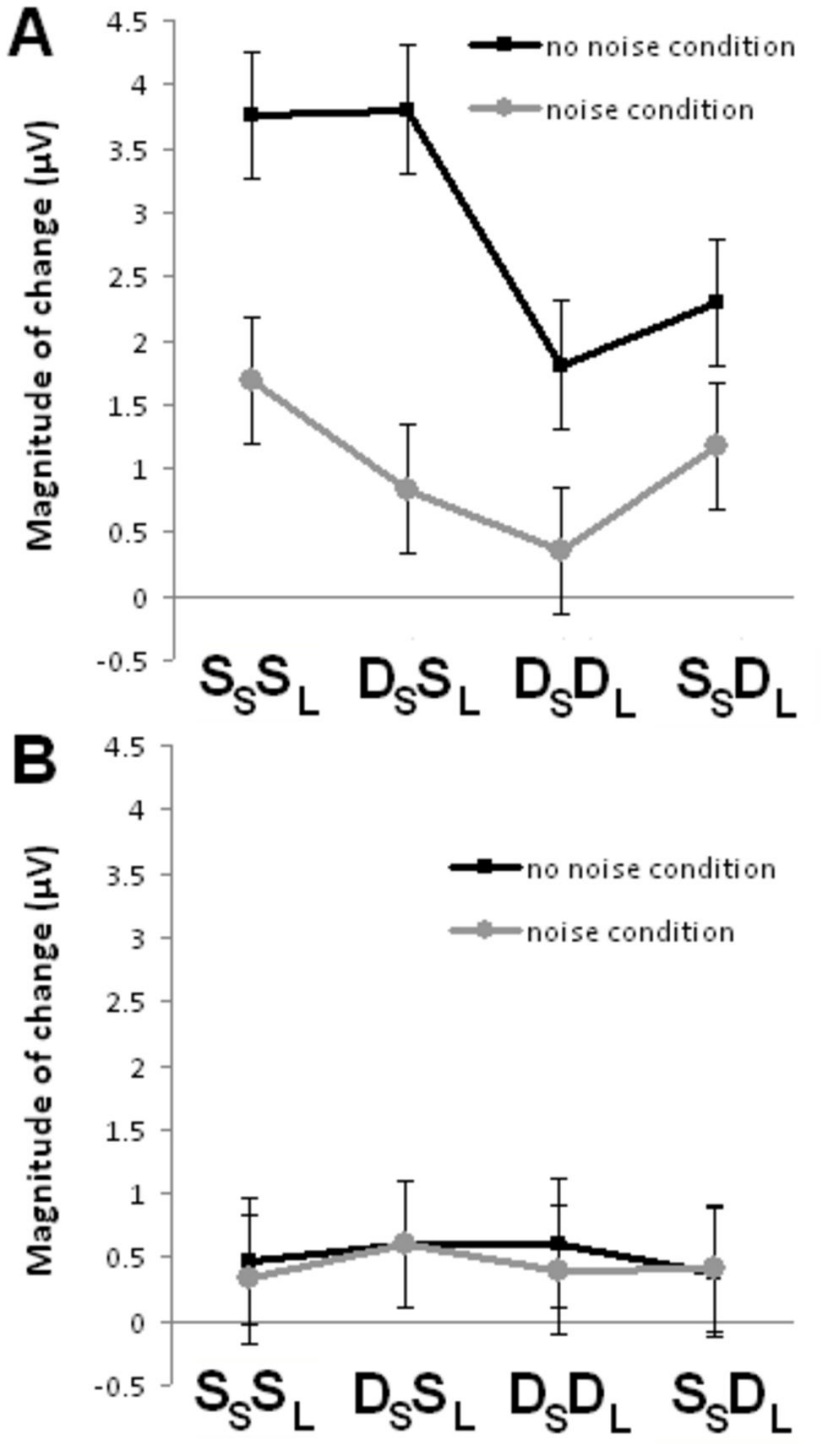
Plot of the magnitude of P2 amplitude changes from the 1^st^ to 4^th^ position across the four trial types in the absence and presence of noise. (A) Young adults. (B) Older adults. Error bars indicate standard error of the mean.

For the P2 wave, repeated measures ANOVA (age × noise condition × trial type) revealed significant main effects of noise condition, *F*(1,22) = 4.918; *p* = 0.037, and trial type, *F*(3,66) = 3.434; *p* = 0.022, and a significant interaction effect between age and trial type, *F*(3,66) = 5.147; *p* = 0.002. In young adults, repeated measures ANOVA revealed a main effect of trial type, *F*(3,66) = 5.786; *p* = 0.003. Pairwise comparison showed that the P2 adaptation was greater for the S_S_S_L_ than the D_S_D_L_ trials and greater for the D_S_S_L_ than the D_S_D_L_ trials (Figure 7A). No significant findings were obtained for older adults, *F*s < 1 (Figure 7B).

## Discussion

### Summary of findings

The present study assessed the effects of age on neural adaptation to real life sounds. We found that the N1 and P2 waves decreased in amplitude with stimulus repetition. This neural adaptation was greater in young than in older adults and was more pronounced when the stimuli were identical within the sequence. Young adults showed neural adaptation in both the N1 and P2 waves but older adults showed much reduced P2 adaptation to any trial type. By analyzing the magnitude of amplitude changes, we found domain-specific adaptation from P2 waves for young adults. Overall, background noise decreased the ERP amplitudes and modulated the magnitude of repetition effects on both the N1 and P2 amplitude (i.e., smaller changes in amplitude from the first to the last sound in the sequence), and this effect was comparable in young and older adults.

### Age differences in adaptation to meaningful sounds

In young adults, stimulus repetition within a four stimulus train was associated with a decrease in N1 and P2 amplitudes, which was more pronounced between the first and second stimuli within the train. This finding replicates and extends previous work on the N1 (e.g., [20,31,34]) and P2 waves (e.g., [11,32,33]) using pure tones. The neural adaptation, especially the reduction of N1 amplitude, which occurred in every trial type, is consistent with habituation and neuronal refractoriness [1,6,35,36]. It has been suggested that neural adaptation reduces the sensitivity of the auditory system to repeated sounds and enhances the sensitivity to novel sounds for learning and memory [3].

In older adults, stimulus repetition generated a decrease in N1 amplitude. The P2 amplitude was little affected by stimulus repetition. While the reduced P2 suppression in older adults is consistent with prior work using brief tones [19], the effects of stimulus repetition on N1 is at odds with studies showing either no or reduced N1 adaption to pure tone stimuli [19,20]. Specifically, the age and position interaction for the N1 amplitude was not significant in the present study. However, Fabiani et al. [20] found an interaction effect between age and position, and older adults showed less suppression in N1 amplitude than young adults across the stimulus train. The difference between our findings and those of prior studies (e.g., [19,20]) could be related to the material used. Pure tone stimuli likely promote frequency-specific neural adaptation, which could be sensitive to aging due to impoverished spectral acuity [37]. The complex sounds used in the present study likely comprise a wide range of frequencies, thereby reducing possible age differences related to spectral acuity and frequency selectivity.

The reduced P2 adaptation for meaningful sounds in older adults may be related to deficits in sensory memory [13,38]. Older adults may have difficulty maintaining an accurate representation of the acoustic details of the incoming stimulus, such that every complex sound in the sequence is essentially treated as “novel” and processed at a further level. Evidence from behavioral and neuroimaging techniques suggest that auditory sensory memory can be divided into at least two stages: one is a short auditory store that extends the apparent duration of a stimulus up to about 300 ms, and the other is a long auditory store that retains auditory information for several seconds, minutes, or hours [39]. The former has been regarded as a short-term mechanism that involves the reverberation of neuronal activity within memory traces in the brain, whereas the latter is more like a long-lasting synaptic change contributing to long-term potentiation [40]. Previous studies have shown that stimulus repetition causes an immediate decrement in neuronal responses that lasts several hundred milliseconds and comprises the memory trace (e.g., [41]). This response decrement appeared to coincide with the P2 latency [11]. Some auditory studies on healthy adults also reported diminished P2 amplitudes with repeated sounds and memory trace formation [34,42]. Based on these findings, the reduced P2 suppression in older adults could reflect an age-related deficit in the early phase of sensory memory.

Another possibility is that aging impedes the listener’s ability to form sound object representations, which could be indexed by the P2 wave. In young adults, memory traces are specific to individual sounds, and therefore repetitive stimulation of specific memory traces by identical stimuli induces suppression of the evoked potentials, with the silent intervals allowing the recovery process to occur [38,40]. Some studies showed that the development of specific memory traces in the human auditory cortex is fast, taking as little as tens of seconds after repetitive stimulation [43,44,45]. In another study, Richardson et al. [46] measured the P3 wave (a late positive deflection that peaks between 200 and 520 ms at parietal sites) elicited by complex, everyday sounds (e.g., dog bark, drum beat and car horn), and found a reduced P3 suppression to sound repetition in older adults compared to young adults. Their results were explained by the fact that older adults were less able to construct category templates [47]. Such deficits in forming category templates could also account for the reduced P2 adaptation that we observed, and appears consistent with prior research showing that categorical perception modulated ERPs at about 200 ms after sound onset [48].

In older adults, the reduced P2 adaptation could also be related to deficits in inhibitory control, including inefficient filtering of repeated information, mediated by the prefrontal cortex [12,13,17,49]. Previous studies have shown that older adults processed repeated irrelevant information more than young adults, which could be due to faster decay of the memory traces [50], or decrease in filtering of irrelevant input [51], or both [52]. In a recent study, Fabiani et al. [20] used pure tones with an onset-to-onset interval of 400 ms, which was empirically tested to produce maximum N1 suppression by the second tone with stimulus repetition (e.g., [53,54]), and found reduced suppression of both N1 and P2 amplitudes in older adults. They suggest that inhibitory processes are reduced or become slower and more sluggish with age. The larger N1 amplitude among the older adults in Fabiani et al.’s study [20] may indicate that attention was captured by the repeated irrelevant stimuli. Our results are in-line with this interpretation as the older adults also showed significantly larger N1 amplitude than young adults throughout the entire stimulus train.

In the present study, stimulus duration was longer than those used in prior studies (e.g., [19,20]). Hence, there is a possibility that the patterns of adaptation effects observed in young and older adults were partly due to this more sustained stimulus presentation. In addition to stimulus duration, some studies reported that an inter-stimulus interval of 10 seconds or less contributed to the adaptation effects [6,55]. As the inter-stimulus interval in the present study was comparatively short, at 295 ms, it was likely that the adaptation effects were a combination of both the repetition of stimuli within the stimulus train and the sustained stimulation of the neural generators.

Prior studies using pure tones have identified several N1 subcomponents based on the waveform latency and scalp distribution [6,56,57]. Among them, the N1a is most prominent at temporal electrodes; the N1b at midline central electrodes and the N1c extends to fronto-polar and right temporal electrodes [6,8]. Despite this distinction, Perrault and Picton [58] were unable to dissociate the N1a from the N1b subcomponent in a variety of experimental manipulations on healthy young adults. It might be due to the fact that these three subcomponents are controlled by the physical and temporal aspects of the stimulus and by the general state of the participants [6]. Others suggest that the major contribution to the auditory N1 suppression effect is circumscribed to late N1 subcomponents such as the N1b and N1c [59]. In the present study, as a mix of complex real life sounds were used, the differences in the physical aspects of the stimulus might cause variability on these N1 subcomponents. It would be interesting, for future studies, to limit the number of meaningful sounds and increase the length of the stimulus train in the experiment, so as to warrant a comprehensive analysis of N1 subcomponents.

### Domain-specific adaptation in young adults

In the present study, domain specific neural adaptation was examined by analyzing the interaction between trial type and position and the magnitude of amplitude change evoked by the first compared to the last sound in the train among the four trial types. For the N1 wave, we found a general decrease in amplitude, which was little affected by the trial type. That is, N1 adaptation was comparable no matter whether the same or different sound was presented at the same or different location. Therefore, in the present study, the N1 wave appears to reflect the general encoding of acoustic energy elicited by sound onset irrespective of the early spectro-temporal details and/or location of the stimulus. The lack of domain-specificity in N1 suppression is consistent with the notion that the N1 wave reflects the registration of sound stimuli and/or signal detection as opposed to signal discrimination [8]. Another possibility is that the complex meaningful sounds used in the present study did not contain sufficient category-specific acoustic details and/or the complex sounds stimulated semantic processing in higher cortical centers that goes beyond early N1 responses [60,61].

The first sign of domain specificity was observed during the P2 wave in young adults. Specifically, we found greater P2 amplitude suppression when sound location was repeated (while sound identity changed) than when sound identity was repeated (while sound location changed). This kind of domain-specific adaptation could be explained by the sharpening model described by Grill-Spector et al. [2]. According to this model, only some of the neurons that initially respond to a stimulus will show repetition suppression to subsequent presentation of the same stimulus. This selective response is thought to be a learning process in which representations are tuned and consequently become sparser, resulting in fewer responsive neurons in total [2,62,63]. In the current study, as only one of the two sound features was repeated, the changes in ERP amplitudes would likely reflect the neuronal response corresponding to the repeating feature. In the event that both features are repeated (i.e., domain-general adaptation), the fatigue model might be a more appropriate model than the sharpening model to explain the mechanism underlying neural adaptation. According to the fatigue model [2], all neurons that are initially responsive to a stimulus will show a proportionally equivalent reduction to their response to repeated presentation of the same stimulus, resulting in a reduction of neuronal firing [2]. This effect was demonstrated in the current study when both sound features were repeated, resulting in a greater extent of neural adaptation for sounds having repetition of both location and identity as compared to sounds comprising different location and spectro-temporal characteristic throughout the trains of stimuli.

In addition to the sharpening and the fatigue models, Grill-Spector et al. [2] also proposed the facilitation model which predicts that repetition causes faster processing of stimuli and results in shorter latencies or shorter durations of neuronal firing. The facilitation model is based on the assumption that synaptic potentiation between neurons following an initial stimulus presentation leads to faster identification of a repeated stimulus [2]. However, this model cannot be used to explain the results of our study as we found no effect on the latencies of the N1 and P2 waves across the stimulus train. In a prior study using pure tones, Zhang et al. [8] found a significant stimulus order effect on the N1 latency, i.e., reducing N1 latencies with progressing stimulus positions along the stimulus train. Their results were indeed consistent with the facilitation model [2]. As mentioned earlier, the discrepancy between our findings and those of prior studies can be accounted for by the material used. For instance, Richardson et al. [46] also reported no effect on ERP latencies with repetition of computer generated novel sounds, like the ones we used here, albeit their focus was on P3 waves. Also, a previous study focusing on neural adaptation to voices did not reveal any effect on N1 and P2 latencies [64]. Another explanation lies in the assumption that the facilitation model is more pronounced with top-down information processing and that the largest effect occurs after initial sensory processing, i.e., after the first 200 ms [2]. As the present study used a passive listening approach where participants listened to the stimuli without the need to give any overt response, the demand for top-down processing was minimal.

In the present study, the P2 wave showed differential patterns of neural adaptation to sound identity and location repetition among young adults. This is in line with visual ERP studies showing that stimulus repetition produced an ERP difference around 150 ms that was greater for repeated visual objects with the same view than with a different view, whereas adaptation for the same object presented at a different field of view was observed subsequently between 400 and 700 ms post-stimulus [65,66].

In the present study, young adults showed greater adaptation to location than identity repetition. This is demonstrated by a greater P2 amplitude decline in location repetition compared to identity repetition. One explanation is that location activates neural responses early in the processing of complex sounds [67], which can lead to more efficient neural adaptation. The neural adaptation to location repetition is thought to involve the inferior parietal and superior frontal regions comprising the dorsal auditory stream [23,68,69,70]. This is consistent with Grady et al.’s [21] fMRI study which found a larger set of brain regions activated for adaptation to location than identity. Another possibility is that the neural adaptation for location repetition is more efficient compared to identity repetition. In a prior ERP study, Teder-Sälejärvi et al. [71] asked young adults to attend selectively to brief tones delivered to different spatial locations and found that attention to sound location is accomplished in two distinct stages, with an initial broadly tuned filtering, followed by a more narrowly focused selection of attended-location deviants. Therefore, adaptation would be more facilitated with location repetition than sound repetition. This might explain why young adults showed a stronger adaptation effect, i.e., a greater P2 amplitude decline, on trials with fixed location (i.e., D_S_S_L_) than those with varied location but identical sounds (i.e., S_S_D_L_) in the present study. Also, as seen in the results, the primary difference between the D_S_S_L_ and S_S_D_L_ trials is the number of stimuli in the stimulus train required to reach maximum suppression. When the location is fixed and the sound identity is changed, only one stimulus repetition is needed to yield maximum P2 suppression, but when the location is changed, two more stimulus repetitions are needed (Figure 5B, D) (It also is worth noting that the P2 amplitude of the D_S_S_L_ trial at the second position was comparable to that of the S_S_D_L_ trial at the fourth position). This faster adaptive response to sound location is in line with the results of Diaconescu et al. [72] who found faster evoked responses to the location compared to the identity condition in a cue-target paradigm. Consequently, one could infer that it would be more advantageous to maintain vigilance to constant sounds that are moving. This perhaps concurs with the evolutionary point of view that keeping track of the direction of sounds is a necessity for survival. For example, an individual has to track the movement of prey and identify whether it is moving towards or away from the individual in a forest. Previous studies have shown that spatial adaptation plays a role in auditory motion processing [9]. Moreover, some studies have reported distinct neural generators for generating evoked response to sound pitch or location [73].

Our results showed no domain-specific adaptation in older adults. This result is consistent with Grady et al.’s [21] study, who reported reduced domain-specific adaptation to location repetitions in the frontal and parietal areas for older adults. As noted in Grady et al.’s study, the reduced repetition effects might be attributed to reduced selectivity of responses across the stimulus train among older adults [74], or reduced inhibitory function in older adults [75]. Similarly, reduced domain-specific adaptation for older adults might be because older adults are more easily distracted by irrelevant sound information [49,76]. In this study, when only location was repeated, older adults were unable to inhibit their response to varied sound content and therefore unable to habituate to location repetition. This in turn led to reduced amplitude decline from either repeated sound location or sound content among older adults.

This study explicitly tested the effect of noise on neural adaptation to everyday sounds. Using ERPs, we found that older adults showed reduced adaptation with or without background scanner noise. This finding indicated that the age-related difference in neural adaptation observed in Grady et al.’s study was not simply due to the noisy MRI environment. The current study compared patterns of neural adaptation between young and older adults and revealed age-related differences in neural adaptation to real life, meaningful sounds, which replicate and extend prior research that used sinusoidal pure tones or harmonic complex sounds. The findings from the present study show that aging not only impairs the encoding of sound features but also appears to impede the formation of sound object representation in sensory memory.

## Conclusions

In this study, we used real life, meaningful sounds and simulated scanner noise, and found neural adaptation in both N1 and P2 waves among young adults but only on N1 waves among older adults. While young adults were able to adapt to identity or location repetition, i.e., domain-specific adaptation, in both the presence and absence of simulated scanner noise, older adults did not seem to adapt as efficiently as young adults. The patterns of neural adaptation were different between young and older adults, with older adults showing smaller amplitude changes than young adults across trains of identical sound stimuli. We also found that only young adults showed domain-specific adaptation on P2 amplitudes. These findings indicate that the neural processes associated with adaptation of meaningful sounds differ between young and older adults.
